# Locality versus globality in bacterial signalling: can local communication stabilize bacterial communities?

**DOI:** 10.1186/1745-6150-5-30

**Published:** 2010-04-27

**Authors:** Vittorio Venturi, Ádám Kerényi, Beáta Reiz, Dóra Bihary, Sándor Pongor

**Affiliations:** 1 International Centre for Genetic Engineering and Biotechnology, 34012 Trieste, Italy; 2 Biological Research Center of the Hungarian Academy of Sciences, H-6701, Szeged, Temesvári krt. 62, Hungary; 3 Institute of Informatics, University of Szeged, Árpád tér 2, H-6720 Szeged, Hungary; 4 Faculty of Information Technology, Pázmány Péter Catholic University, Práter u. 50/a. 1083 Budapest, Hungary

## Abstract

**Background:**

Microbial consortia are a major form of life; however their stability conditions are poorly understood and are often explained in terms of species-specific defence mechanisms (secretion of extracellular matrix, antimicrobial compounds, siderophores, etc.). Here we propose a hypothesis that the primarily local nature of intercellular signalling can be a general mechanism underlying the stability of many forms of microbial communities.

**Presentation of the hypothesis:**

We propose that a large microbial community can be pictured as a theatre of spontaneously emerging, partially overlapping, locally recruited microcommunities whose members interact primarily among themselves, via secreted (signalling) molecules or cell-cell contacts. We hypothesize that stability in an open environment relies on a predominantly local steady state of intercellular communication which ensures that i) deleterious mutants or strains can be excluded by a localized collapse, while ii) microcommunities harbouring useful traits can persist and/or spread even in the absence of specific protection mechanisms.

**Testing the hypothesis:**

Some elements of this model can be tested experimentally by analyzing the behaviour of synthetic consortia composed of strains having well-defined communication systems and devoid of specific defence mechanisms. Supporting evidence can be obtained by *in silico *simulations.

**Implications of the hypothesis:**

The hypothesis provides a framework for a systematic comparison of bacterial community behavior in open and closed environments. The model predicts that local signalling may enable multispecies communities to colonize open, structured environments. On the other hand, a confined niche or a host may be more likely to be colonized by a bacterial mono-species community, and local communication here provides a control against spontaneously arising cheaters, provided that survival depends on cooperation.

**Reviewers:**

This article was reviewed by G. Jékely, L. Aravind and E. Szathmáry (nominated by F. Eisenhaber)

## Background

Many bacteria and other unicellular organisms live in large, multispecies communities in which the participants jointly exploit the resources. Multispecies consortia are a major form of bacterial life and often contain hundreds of different species that share secreted materials in a densely packed environment. Social behaviour must be an essential trait throughout bacterial evolution [1], however there is no sufficient experimental evidence to explain why such consortia can be stable against environmental challenges or against the emergence of non-cooperating cheater mutants. Current explanations suggest that specific, species-dependent defence mechanisms or simple mechanical protection present in particular communities (such as seen in biofilms) may be the major factors underlying the stability [2-4]. One of our motivations is to look for alternative, more general explanations.

Many prokaryotes possess inter-cellular signalling systems which allows species to colonise new habitats, to invade hosts and to spread over surfaces [5-7] A typical example is quorum sensing (QS) which enables bacteria to switch from low activity to high activity regimes using signalling molecules as well as various public goods (e.g. surfactants, enzymes, siderophores) that facilitate movement, nutrient uptake amongst other things [7,8]. Signalling molecules are believed in most cases to be transferred by diffusion in the medium surrounding the bacterial populations so their local concentration can vary according to local cell density, positional and/or spatial constraints (for a recent review see [9]). It is also known that a large part of sequenced bacterial species contain sensors for exogenous signals produced by other bacteria or potential host organisms [10,11]. In line with our reasoning is that intercellular signalling may be in part responsible for the stability of bacterial consortia.

Recently we presented an agent based model of bacterial quorum sensing [12] in which the bacterial agents randomly moving on semi-solid agar surfaces, in a process called swarming [13]; in this scenario, bacteria are communicating via diffusible molecules (in this case the signals were *N*-acyl-homoserine lactones). In this model, food is converted into diffusible molecules, cellular motion as well as stored energy that ultimately fuels division of the cells. According to this model, swarming bacterial models were seen to spontaneously form an "active zone", a well-defined region of the surface, in which both diffusible molecules and bacterial cells were present in sufficient quantities so as to maintain the community in a quasi steady state. In this state, cell division must compensate or exceed the death rate and production of chemical signals and public goods must compensate for the losses caused by diffusion and decay. This situation is analogous to (quasi) steady states observed in many physical, chemical and social systems[14,15]. It is worth noting that our model is one in a long series of agent-based models in which intercellular communication is primarily local, i.e. it is directed to a finite neighbourhood around each agent [16-20]. In line with the above, we recently demonstrated that collapse of a swarming community can localize cheater mutants [21]. This leads us to the hypothesis that local communication, hence local community formation may be one of the driving forces responsible for the stability of bacterial consortia. More specifically we suggest that local communication gives rise to a quasi steady state of cooperative exchanges, and the kinetics of cooperation provides protection to bacterial consortia.

## Presentation of the hypothesis

**Figure 1:**
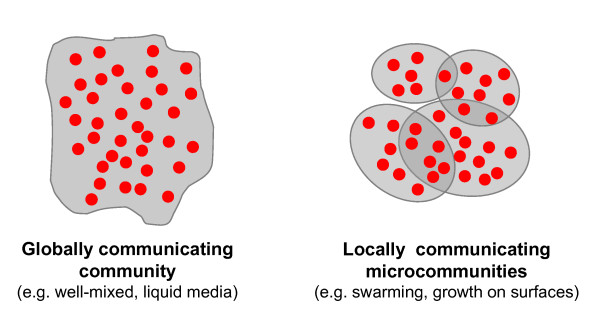
**The local communication hypothesis (right) stresses that members of microbial communities communicate locally, so only locally recruited microcommunities are in close contact**. Microcommunities are defined in an overlapping fashion so that signals will eventually reach all members of the community. The hypothesis predicts that local communication conveys stability against cheater mutants via a local collapse of the affected microcommunities that does not necessarily spread to the entire colony. The cells are denoted by red dots, the gray contour indicates the boundaries of communication.

**Figure 2:**
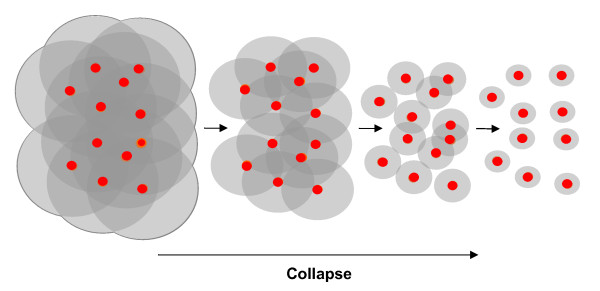
**A microcommunity (left, marked by grey contour) collapses when communication among the members decreases below a critical level**. This can result from decreased signalling or from a decreased density of signal emitting cells, which can be caused, for instance, by a shortage or overconsumption of resources (nutrients, public goods), by an excess of non-communicating cells (cheaters) or by the cells drifting away from each other.

We propose that a large microbial community can be pictured as a theatre of spontaneously emerging, partially overlapping, locally recruited microcommunities whose members communicate primarily among themselves via secreted molecules or cell-cell contacts (Figure 1, right). Specifically, we hypothesise that local communication provides stability by ensuring that i) arising non-cooperating mutants or incoming non-cooperating strains will cause local collapse in the affected microcommunity (Figure 2), which will preclude the spread of the non-cooperators to the whole population, and ii) efficient co-operators can self-organize into microcommunities that can spread better than the rest of the population. We propose that this mechanism is important in open environments where survival depends on a (quasi) steady state of cooperation. In closed and well-mixed homogenous systems, such as laboratory liquid bacterial cultures, interactions are global, so cheaters cannot be isolated and eliminated by local collapse [22,23].

We therefore propose that local bacterial communication within large consortia ensures that microcommunities are recruited and eliminated locally and this phenomenon conveys a measure of kinetic stability to microbial consortia even in the absence of specific mechanisms (antibiotics, extracellular matrices etc.).

## Testing the hypothesis

Some elements of this model can be tested experimentally by comparing the behaviour of synthetic consortia living in open and closed environments. We suggest that construction of strains having well-defined communication systems and devoid of specific defence mechanisms can provide suitable model systems to test the hypothesis on smaller consortia containing a few species. Swarming communities of bacteria, such as *Pseudomonas aeruginosa *[13], *Serratia sp *[24], *Proteus mirabilis *[25] etc. appear to be especially useful models since their signalling systems are well understood [26-28]. The behaviour of large, multispecies communities may be a challenge since some of the participating species will inevitably contain specific mechanisms that may complicate the interpretation of the results. Supportive evidence can be collected for instance via the study of mutation rates: communities dependent on cooperation in an open environment should have a smaller number of mutants as compared to the same community in a well-mixed (i.e. globally communicating) environment. Another experiment might include the study of mutants varying in terms of fitness. Our hypothesis predicts that greedier, faster dividing mutants that do not contribute to the production of public goods are more likely to collapse a community than mutants that divide only slightly faster than the wild type cells.

## Implications of the hypothesis

Explaining the stability and resilience of biological communities has been a long-standing aspiration from both theoretical and clinical perspectives. Our hypothesis is meant to point out that stability of microbial communities can be formulated in terms of an exchange of signals and secreted molecules, and the local nature of the communication provides a measure of stability to the community. In contrast to specific defense mechanisms, the present hypothesis is not concerned with the species-specific properties, thus it can be applied also to multispecies consortia. We think that the general mechanism outlined here complements rather than replaces other known, specific mechanisms.

The present hypothesis refers to an exchange of molecules and/or signals between bacteria which is an *ad hoc *short term event. In this sense, we consider a community stable at the level of functional repertoire if it collectively possesses the traits necessary to survive in a given environment. On the other hand, a community can be also considered stable at the metagenomic level if its members collectively contain a certain set of genes sufficient to ensure the survival of the community. However, the hypothesis is not meant to explain the evolutionary fate of the underlying mechanisms and/or the evolution of cooperation (an excellent overview is in [29]).

The hypothesis provides a framework in which the stability of communicating cellular communities (bacterial consortia, host-pathogen ensembles etc.) can be approached and new clinical intervention strategies can be formulated. For instance, QS has been implied as the mechanism by which bacteria establish themselves in their hosts, and QS mutants have been suggested as suitable tools of intervention that can interfere with the infectivity of pathogenic bacteria by competing out wild type pathogens within the host [30]. On the other hand, our hypothesis suggests that QS mutants may not be necessarily able to overtake an entire habitat, if cooperation is necessary for colonizing the host organism.

Our hypothesis does not imply that microbial communication is exclusively local, we rather suggest that local interactions dominate some of the essential features of the community. We simply imagine bacterial communities to have a balance of local and global communication in which interactions within distant cells are not as frequent as those within immediate neighbours. This can be pictured as a colony being composed of many overlapping communities defined by their communication zone of intercellular signalling. As this radius increases, communication becomes increasingly global. For instance, contact-based signalling between cells [31] is more local than communication via diffusible signals. If communication breaks down between parts of the community, the group will split into distinct parts, such as may be the case when a part of the community collapses, or when a microcommunity breaks away from the rest of the colony. Also in the predominantly local interactions, there is a measure of globality in as much as the signal can be passed from neighbour to neighbour.

The hypothesis provides a framework for systematic comparison of bacterial community behavior in open and closed environments. The model predicts that local signalling may enable multispecies communities to colonize open, structured environments by putting a security control in place against non-cooperators/cheaters. On the other hand, a relatively closed/confined niche or a host may be more likely to be colonized by a bacterial mono-species community, and local communication here provides a control against spontaneously arising cheaters.

It was pointed out by one of the reviewers of this paper that the local communication/local interactions hypothesis does not rely on community properties that are exclusively bacterium-specific (E. Szathmáry, personal communication). This makes us speculate that a similar reasoning may be applicable to other systems that can be modelled as ensembles of locally interacting entities. Protein structures, protein assemblies can all be pictured as networks dominated or at least influenced by local interactions, and recruitment of molecular assemblies or the overlapping module-structure of protein interaction networks provide further intriguing analogies. Nevertheless, the interpretation of locality and globality may greatly vary from system to system, so such wide analogies need to be interpreted with care.

What is then the difference between the present hypothesis and the current views on biological communities? Current paradigms of biological communities use concepts such as altruism, mutuality, kinship, policing, spite, etc., i.e. phenomenological notions that stress the analogies with social sciences and game theory. In contrast, the key concepts of the local communication hypothesis are diffusion, steady state and signalling, i.e. terms related to an underlying mechanism, borrowed from physicochemistry and information technology. We think the two views complement rather than exclude each other.

## Competing interests

The authors declare that they have no competing interests.

## Authors' contributions

VV and SP conceived the study.  AK, BR and DB performed modelling experiments and helped to draft the manuscript. All authors read and approved the final manuscript.

## Reviewers' comments

### Reviewer 1: L. Aravind, National Center for Biotechnology Information, National Library of Medicine, National Institutes of Health, Bethesda, MD 20894

**Reviewer's comment**: In recent years there is mounting evidence that the behavior and biochemistry of bacterial communities have key lessons for understanding diverse evolutionary processes. A key behavioral problem is how diverse bacterial communities show resilience in face of cheaters that maximize their own fitness at the cost of other members of the community. Biochemical evidence, supported by genomics, suggests that several measures such as lineage-specific diversification of siderophores (e.g. the pyoverdine operon in Pseudomonas), and use of mechanical protection as in biofilms might be defense mechanisms of communities. However, it is not clear if these specific defenses are the only explanations for the problem under consideration. The current article proposes a different kind of hypothesis invoking more a general process to explain resistance to destructive behaviors. The current hypothesis is certainly amenable to different kinds of tests and qualifies as a valid proposal in this regard. A more difficult question is how the proposed process interacts with the other more specific explanations. This includes questions such as: Do the specific and general mechanisms act in the same conditions or are they approaches specific to certain environmental conditions? For example, it is possible that the general mechanism dominates in solid substrates such as soil whereas the specific mechanisms are more valid in bulk aqueous media. Again, a specific mechanism such as the formation of an extracellular matrix could potentially be a facet of the more general mechanism proposed in this article.

**Authors' response**: We speculate that the mechanisms of local collapse/local communication play a role in heterogeneous, open systems and that they cannot play a role in perfectly mixed, homogenous media. In the latter conditions only the specific mechanisms are likely to play a role. We considered extracellular matrix formation as a specific defense mechanism, however it is true that it also constrains the diffusion of secreted materials. These points are now addressed in the text.

**Reviewer's comment**: An optional suggestion to the authors: A schematic figure summarizing the hypothesis might be helpful for the reader to get a quick over view.

**Authors' response**: We have now included two explanatory figures.

### Reviewer 2: Gáspár Jékely, Max Planck Institute for Developmental Biology, Tübingen, Germany

**Reviewer's comment**: In this paper Venturi et al[21] present a hypothesis that the primarily local nature of intercellular communication in microbioal communities is a source of stability. This is an interesting idea and the hypothesis could stimulate experimental work to test its predictions.

The authors start to present their hypothesis by stating that "We propose that intercellular communication in bacterial consortia is primarily local". I think that the local nature of intercellular communication should not be presented as a part of their hypothesis, but as a known fact arising from physical constraints (diffusion constants etc.). To make this point first, the authors should briefly discuss what is known about the diffusion of various molecules in bacterial communities. A relevant paper about the diffusion of N-acyl-homoserine lactones in biofilms for example is Alberghini et al. FEMS Microbiol Lett. 2009 Mar;292(2):149-61 [9].

**Authors' response**: We fully agree that the local nature of AHL diffusion is not part of the hypothesis. We have moved this point to the Background section along with the suggested discussion on diffusion.

**Reviewer's comment**: In the Background section the author write: "Multispecies consortia often contain hundreds of different species that share secreted materials in a densely packed environment." It would be of course over-ambitious to explain this phenomenon with cooperation and a single hypothesis. Other factors certainly also contribute to the formation of such multispecies consortia. One can for example imagine a mutualistic, syntrophic relationship among several species, where some consume the metabolic waste products of others. These kinds of associations are not endangered by cheaters. Could these maintain the consortia? Maybe. What these interactions cannot explain is the secreted "public goods". This is a cooperative strategy that can be exploited by cheaters. But it is possible that only a few species cooperate, and the others live on them somehow (without necessarily exploiting them). These possibilities, and what the hypothesis tries to explain, could be introduced more carefully.

**Authors' response**: It was not our intention to imply that local communication is the only mechanism that can contribute to the stability of a community. This is now pointed out in the text. On the other hand, we think it is possible that other forms of coexistence and mutualism can be abused by non-cooperators, and if the coexistence is mediated by an exchange of diffusible materials, the stabilizing effect will be similar to what we suggest for QS bacteria.

**Reviewer's comment**: The part on the experimental validation of the hypothesis is relatively vague. It would be helpful to give a few concrete examples, with more literature cited, where this problem can be tested experimentally.

**Authors' response**: We added a few examples to the corresponding paragraph.

**Reviewer's comment**: (Minor comment) Figure 2 depicts the collapse of a microcommunity. It would be more illustrative to draw it together with neighbouring, non-collapsing microcommunities.

**Authors' response**: This is now done.

### Reviewer 3: Eörs Szathmáry, Collegium Budapest, 1014 Hungary, nominated by F. Eisenhaber

**Reviewer's comment**: Large, multispecies communities are a dominant form of life in the microbial world, so understanding their stability is fundamentally important. Environmental challenges, invasion by non-compatible organisms or the emergence of deleterious mutations can all disrupt stable cooperations so microbial communities have to be resilient against all of these factors. Current explanations are usually based on specific defence mechanisms that respond to individual types of challenges. The paper of Venturi et al. [21] develops a general scenario in which the localized nature of intercellular communication is used to explain the stability of bacterial communities. In contrast to specialized, species-specific mechanisms, this explanation is supposed to be common to all communities that need to cooperate in order to survive. It is not clear from the description if this hypothesis suggests different scenarios for monospecies and multispecies communities, it appears that it is applicable to both. Also, even though quorum sensing is a central example in this paper, the hypothesis apparently does not rely on specific bacterial features, so it may be applicable to other communities and ensembles.

**Authors' response**: In fact, the hypothesis can be applied to any ensemble of interacting entities that communicate locally, and as a consequence, the hypothesis can be applied to both monospecies and multispecies communities, provided that the members of the community can collectively secure the functional repertoire necessary for survival.

**Reviewer's comment**: Finally the authors may want to explain the relation of this supposed general mechanism to the body of literature on specific defense mechanisms. Also, it is worthwhile to look at modelling approaches that embrace components of cooperation and communication. In particular, the paper by Czárán and Hoekstra (Microbial Communication, Cooperation and Cheating: Quorum Sensing Drives the Evolution of Cooperation in Bacteria [29]) requires special attention.

**Authors' response**: This is now done in the Implication and Conclusions section of the manuscript.

**Reviewer's comment**: As a more general remark, one should appreciate that distinction should be made between situations that are covered by weak altruism (where the altruist pays only a relative fitness cost) and those that rest on strong altruism. Only the latter requires kin selection, although for multispecies communities the latter is not a trivial mechanism to follow. Some discussion of this issue seems in order.

**Authors' response**: This is a loaded question and we feel that a full discussion would be beyond the scope of this work. According to our prelimiary modelling studies, certain types of cheater mutants, with the slightest fitness advantage are capable to produce a local collapse, however we feel that the evaluation of fitness in an open, heterogeneous environments may require a more careful studies.

Finally we thank all three reviewers for the useful and thought-provoking comments.

## References

[B1] Maynard Smith J Szathmáry E The Major Transitions in Evolution 1995 Oxford University Press

[B2] Mah TF O'Toole GA Mechanisms of biofilm resistance to antimicrobial agents Trends Microbiol 2001 9 34 39 10.1016/S0966-842X(00)01913-2 11166241

[B3] O'Toole GA Microbiology: a resistance switch Nature 2002 416 695 696 10.1038/416695a 11961541

[B4] O'Toole GA Stewart PS Biofilms strike back Nat Biotechnol 2005 23 1378 1379 10.1038/nbt1105-1378 16273068

[B5] Bassler BL Small talk. Cell-to-cell communication in bacteria Cell 2002 109 421 424 10.1016/S0092-8674(02)00749-3 12086599

[B6] Camilli A Bassler BL Bacterial small-molecule signaling pathways Science 2006 311 1113 1116 10.1126/science.1121357 16497924 PMC2776824

[B7] Fuqua C Parsek MR Greenberg EP Regulation of gene expression by cell-to-cell communication: acyl-homoserine lactone quorum sensing Annu Rev Genet 2001 35 439 468 10.1146/annurev.genet.35.102401.090913 11700290

[B8] Fuqua C Greenberg EP Listening in on bacteria: acyl-homoserine lactone signalling Nat Rev Mol Cell Biol 2002 3 685 695 10.1038/nrm907 12209128

[B9] Alberghini S Polone E Corich V Carlot M Seno F Trovato A Squartini A Consequences of relative cellular positioning on quorum sensing and bacterial cell-to-cell communication FEMS Microbiol Lett 2009 292 149 161 10.1111/j.1574-6968.2008.01478.x 19187204

[B10] Ryan RP Dow JM Diffusible signals and interspecies communication in bacteria Microbiology 2008 154 1845 1858 10.1099/mic.0.2008/017871-0 18599814

[B11] Subramoni S Venturi V LuxR-family 'solos': bachelor sensors/regulators of signalling molecules Microbiology 2009 155 1377 1385 10.1099/mic.0.026849-0 19383698

[B12] Netotea S Bertani I Steindler L Venturi V Pongor S A simple model for the early events of quorum sensing in *Pseudomonas aeruginosa* Biol Direct 2008 4 6 10.1186/1745-6150-4-6 PMC2660287 19216743

[B13] Daniels R Vanderleyden J Michiels J Quorum sensing and swarming migration in bacteria FEMS Microbiol Rev 2004 28 261 289 10.1016/j.femsre.2003.09.004 15449604

[B14] Daly H Steady-State Economics 1991 2 Washington DC: Island Press

[B15] Wimpenny JWT Kinniment S Lappin-Scott HM, Costerton JW Biochemical Reactions and the Establishment of Gradients within Biofilm Microbial Biofilms 2003 1 Cambridge: Cambridge University Press 99 117

[B16] Ben-Jacob E Schochet O Tenenbaum A Cohen I Czirok A Vicsek T Generic modelling of cooperative growth patterns in bacterial colonies Nature 1994 368 46 49 10.1038/368046a0 8107881

[B17] Cohen I Mathematical Modeling and Analysis of Pattern Formation and Colonial Organization in Bacterial Colonies 2006 Tel-Aviv University, Department for Applied Mathematics

[B18] Gerlee P Anderson AR A hybrid cellular automaton model of clonal evolution in cancer: the emergence of the glycolytic phenotype J Theor Biol 2008 250 705 722 10.1016/j.jtbi.2007.10.038 18068192 PMC2846650

[B19] Kawasaki K Mochizuki A Matsushita M Umeda T Shigesada N Modeling spatio-temporal patterns generated by Bacillus subtilis J Theor Biol 1997 188 177 185 10.1006/jtbi.1997.0462 9379672

[B20] Reynolds CW Flocks, herds and schools: a distributed behavioral model Computer Graphics 1987 21 25 34 10.1145/37402.37406

[B21] Venturi V Bertani I Kerényi Á Netotea S Pongor S Co-swarming and local collapse: quorum sensing conveys resilience to bacterial communities by localizing cheater mutants in Pseudomonas aeruginosa PLoS One 2010 5 e9998 10.1371/journal.pone.0009998 20376321 PMC2848674

[B22] Diggle SP Griffin AS Campbell GS West SA Cooperation and conflict in quorum-sensing bacterial populations Nature 2007 450 411 414 10.1038/nature06279 18004383

[B23] Sandoz KM Mitzimberg SM Schuster M Social cheating in *Pseudomonas aeruginosa *quorum sensing Proc Natl Acad Sci USA 2007 104 15876 15881 10.1073/pnas.0705653104 17898171 PMC2000394

[B24] Eberl L Molin S Givskov M Surface motility of *Serratia liquefaciens *MG1 J Bacteriol 1999 181 1703 1712 10074060 10.1128/jb.181.6.1703-1712.1999 PMC93566

[B25] Gibbs KA Urbanowski ML Greenberg EP Genetic determinants of self identity and social recognition in bacteria Science 2008 321 256 259 10.1126/science.1160033 18621670 PMC2567286

[B26] Smith RS Iglewski BH P. aeruginosa quorum-sensing systems and virulence Curr Opin Microbiol 2003 6 56 60 10.1016/S1369-5274(03)00008-0 12615220

[B27] Van Houdt R Givskov M Michiels CW Quorum sensing in Serratia FEMS Microbiol Rev 2007 31 407 424 10.1111/j.1574-6976.2007.00071.x 17459113

[B28] Venturi V Regulation of quorum sensing in Pseudomonas FEMS Microbiol Rev 2006 30 274 291 10.1111/j.1574-6976.2005.00012.x 16472307

[B29] Czaran T Hoekstra RF Microbial communication, cooperation and cheating: quorum sensing drives the evolution of cooperation in bacteria PLoS One 2009 4 e6655 10.1371/journal.pone.0006655 19684853 PMC2722019

[B30] Rumbaugh KP Diggle SP Watters CM Ross-Gillespie A Griffin AS West SA Quorum sensing and the social evolution of bacterial virulence Curr Biol 2009 19 341 345 10.1016/j.cub.2009.01.050 19230668

[B31] Igoshin OA Mogilner A Welch RD Kaiser D Oster G Pattern formation and traveling waves in myxobacteria: theory and modeling Proc Natl Acad Sci USA 2001 98 14913 14918 10.1073/pnas.221579598 11752439 PMC64958

